# Incidental Findings of Asystole in a Patient With Complaints of Near Syncope: A Case Report on Paroxysmal Ventricular Standstill

**DOI:** 10.7759/cureus.18438

**Published:** 2021-10-02

**Authors:** William J Moles, Anne A Barnes, Ahmed Khan, Kashyap Patel, Nadine Bos

**Affiliations:** 1 Internal Medicine, Edward Via College of Osteopathic Medicine, Salem, USA; 2 Internal Medicine, Lewis Gale Medical Center, Salem, USA; 3 Internal Medicine, Graduate Medical Education, Lewis Gale Medical Center, Salem, USA

**Keywords:** advanced heart block, ventricular standstill, asystole, cardiology research, life threatening arrhythmia

## Abstract

Paroxysmal ventricular standstill (PVS) is an unusual cardiac phenomenon in which the heart experiences episodes of absent ventricular activity despite normal atrial functioning, often leading to cardiac arrest and syncope. In this case, we report the hospital stay of a 70-year-old male who was admitted to the hospital following an episode of near syncope at home. On admission, the patient’s initial electrocardiogram (ECG) showed sinus rhythm at 60 beats per minute without atrioventricular (AV) block. However, as orthostatic vitals were obtained, the patient became lightheaded for several seconds upon standing, which was noted to correspond with a nine second episode of asystole on telemetry and spontaneous return to sinus bradycardia afterward. Cardiology was immediately consulted and confirmed the diagnosis of paroxysmal ventricular standstill (PVS). Given continued episodes of PVS, the patient underwent successful urgent dual-chamber pacemaker placement, following which he became asymptomatic with resolution of bradycardia. Given the high mortality risk associated with PVS, this condition is an important differential to consider in any patient presenting with syncope or near syncope of unclear etiology.

## Introduction

Paroxysmal ventricular standstill (PVS) is a rare electrophysiologic abnormality characterized by periods of complete atrioventricular (AV) block without any ventricular escape rhythm. The term was coined in 1952 by Harold Cookson, when he reported three cases of the disease [1]. During episodes of PVS, the sinoatrial node is firing and atrial depolarization is occurring, however, there is no ventricular activity to provide any cardiac output. These events can last for seconds at a time, often resulting in a syncopal event that is about ten times as deadly as ventricular fibrillation [2]. Treatment includes immediate cardiopulmonary resuscitation and urgent permanent pacemaker insertion.

## Case presentation

A 70-year-old male with a past medical history of aortic stenosis, hypertension, type 2 diabetes mellitus, dyslipidemia, and benign prostatic hypertrophy presented to the emergency room following a near syncopal episode and fall at home. He described the event as an episode of dizziness and denied any loss of consciousness, palpitations, or chest pain. Although the fall was unwitnessed, the patient’s wife claimed that the patient was fully alert and oriented upon assisting him within one minute of the fall. Of note, the patient did endorse increasing fatigue over the past month but denied any syncope or near syncopal events prior to this episode.

The patient was on semaglutide and glimepiride for diabetes and had experienced two hypoglycemic episodes in the past year, however, he stated that this event felt different, and he endorsed no resolution of symptoms after taking a glucose pill. The patient had been on doxazosin 4 mg for several years for the treatment of his benign prostatic hyperplasia without any complaints. Notably, the patient was not on any heart rate controlling medications. 

Upon further investigation, it was discovered that the patient had undergone a treadmill stress test two weeks prior as part of presurgical clearance for a noncardiovascular procedure. The patient experienced no angina during the test, and serial electrocardiograms (ECGs) revealed no ST or T wave abnormalities or arrhythmias. Previous echocardiograms were unable to be obtained as the patient endorsed not having seen a cardiologist in at least seven or eight years due to lack of cardiovascular-related symptoms. The patient did have a coronary artery catheterization performed in 2003 for a complaint of chest pain, which revealed a dominant circumflex system and a normal left ventricular function with no coronary artery disease. No further workup was done regarding this event.

On admission, the patient was hemodynamically stable with a baseline heart rate between 50 and 60 beats per minute (bpm). Physical exam was normal except for a 2/6 systolic ejection murmur at the right upper sternal border. The patient was awake, alert, and oriented during the admission interview. Initial electrocardiogram (ECG) showed sinus rhythm at 60 bpm with mild left axis deviation and no AV block. Laboratory testing revealed a troponin I of 0.01 ng/dl, pro-B-type natriuretic peptide (Pro-BNP) 54 pg/ml, thyroid-stimulating hormone (TSH) 1.0 mIU/l, glucose 117 mg/dl, and electrolytes within normal limits. 

**Figure 1:**
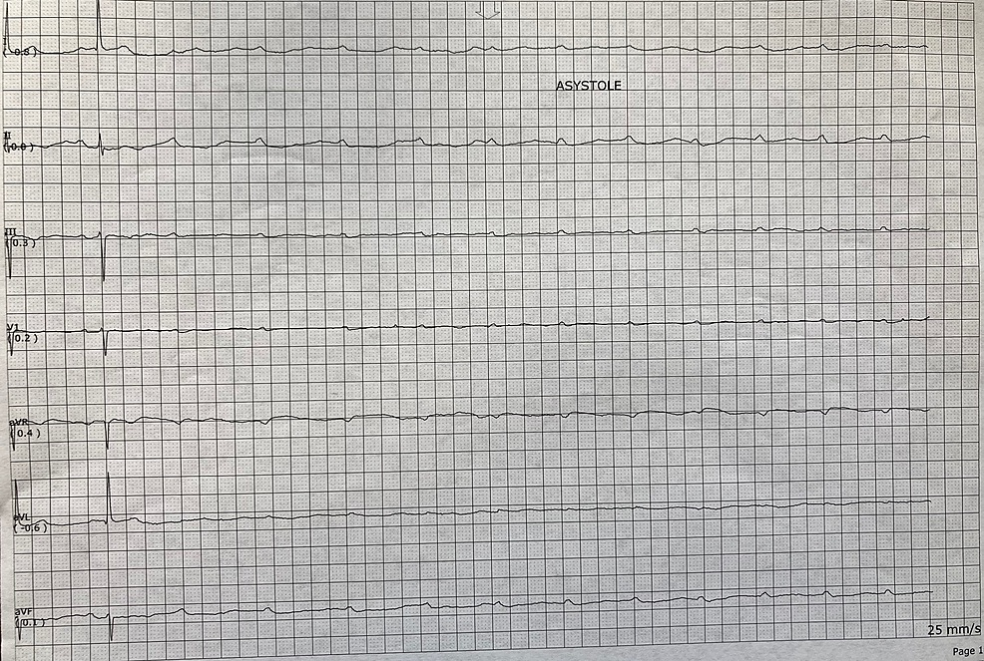
Telemetry strip recording a nine-second period of PVS. PVS: paroxysmal ventricular standstill.

**Figure 2:**
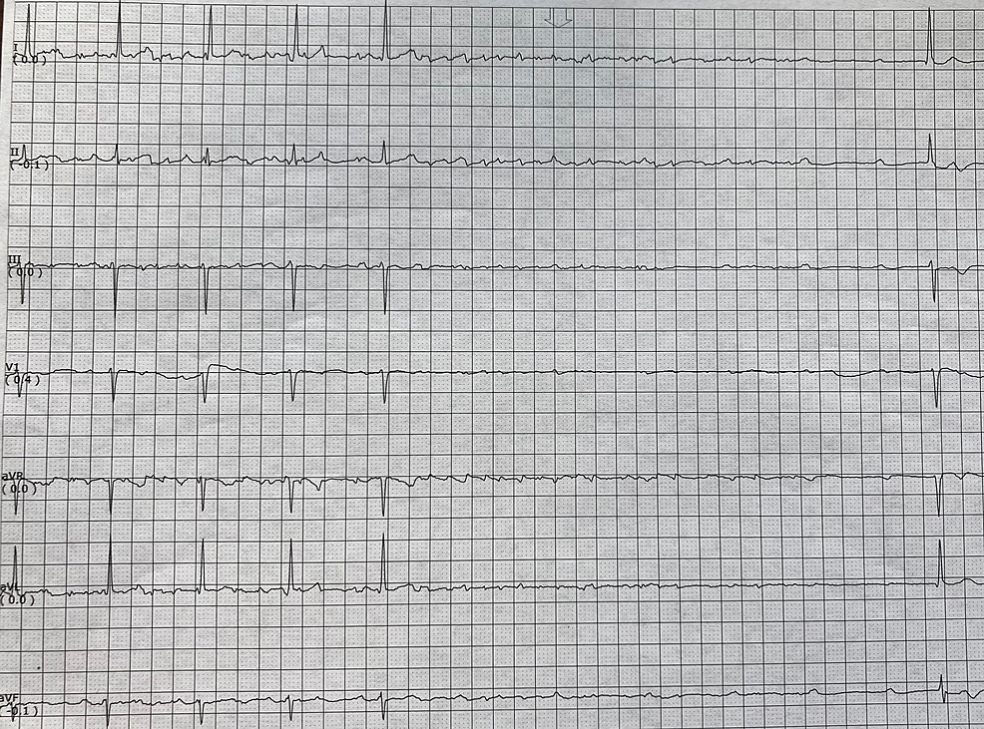
Telemetry strip recording a five-second period of PVS. PVS: paroxysmal ventricular standstill.

During orthostatic testing of the patient, the nurse noticed the patient became lightheaded upon standing for a period of 10-15 seconds before returning to baseline. This period corresponded with a telemetry reading of asystole for nine seconds (Figure 1). Upon further evaluation, the telemetry strip revealed the presence of P waves with total absence of corresponding QRS complexes. After this nine-second period, the rhythm spontaneously returned to a normal 1:1 P to QRS conduction with a normal P-R interval and a heart rate of 50 bpm. Cardiology was immediately consulted and diagnosed the patient with PVS. Repeat ECG at this time revealed sinus bradycardia at 53 bpm with no AV block. During cardiology’s evaluation of the patient, he underwent another five seconds of PVS, this time with the spontaneous return to sinus bradycardia at 30-35 bpm with no AV block (Figure 2). At this point, it was decided that the patient should undergo an urgent dual-chamber pacemaker placement, following which he became asymptomatic with a heart rate of 70-80 bpm.

## Discussion

Paroxysmal ventricular standstill is a rare phenomenon with an unclear prevalence. The scarcity of data pertaining to PVS can be attributed to both the lack of well-documented cases of the disease, as well as the fact that PVS has often masqueraded as other phenomena. For instance, there are several recorded cases in which PVS associated syncope was originally misdiagnosed as primary epilepsy due to the myoclonic and generalized convulsive activity that can manifest in prolonged episodes [3,4]. 

The exact mechanism of PVS has not been fully elucidated in the literature, however, it appears to be related to a breakdown in the atrioventricular conduction system. In a case series conducted by Parkinson et al. during which 33 instances of PVS were examined, only one case revealed a patient with normal rhythm and intact AV conduction between episodes [5]. In addition, the vast majority of cases in this series had multiple syncopal or near-syncopal events prior to hospital presentation. 

Many cases in the literature readily identify secondary causes of PVS. One report details a patient with a predisposing lesion in his cardiac conduction system along with carotid sinus pressure that ultimately resulted in an attack [6]. Several others describe PVS in patients with vagal hypertonicity from intractable vomiting [7,8]. In the case we present, there was an absence of such an obvious source, and electrocardiography studies surrounding the episodes were largely unremarkable. The patient’s tracings immediately prior to and following both PVS events reveal normal P-R intervals with 1:1 P to QRS conduction, which is surprising considering the length of standstill. Even in the presence of significant bradycardia upon returning to sinus rhythm, these findings, along with a recently unrevealing ECG stress test, contradict a typical picture of advanced heart block. Admittedly, the patient’s subjective sense of fatigue over the previous month, along with the fact that he responded positively to permanent pacing, does lend clinical evidence to some level of preexisting AV nodal dysfunction. However, the lack of anginal symptoms or syncopal events prior to the first instance of near syncope in the shower is an atypical picture even for PVS. In most documented cases, there is an insidious symptom course characterized by intermittent anginal events and attacks of unconsciousness [5].

There are a few reports of PVS-like our case, where AV dissociation was not visualized on ECG and a source of heart block was not readily apparent. For example, You et al. published a case report in 2007 of a 62-year-old male with dizziness and suspected generalized seizures that were later diagnosed as attacks of PVS. In this instance, electrocardiography in between episodes revealed sinus rhythm and an absence of AV block [4]. Mu et al. in 2019 described a case of a 70-year-old male with a syncopal episode, who had an initially normal ECG that was later recognized as PVS [9]. To better comprehend the discrepancy between the electrophysiology data and the symptomatology that is detailed in these two cases, as well as our own, we need a more definitive pathophysiologic mechanism for PVS. Cookson et al., in his article where he coined the term PVS, proposed the notion that AV block is not always a readily identifiable phenomenon on ECG or telemetry because it is not always permanent. He suggests that the cardiac rhythm can rapidly pass back and forth from normal to advanced AV block within a fraction of a second [1]. This is one of the only theories in the literature attempting to explain this atypical presentation of PVS, and while it certainly accounts for the findings in our patient, it ultimately lends little insight into what is occurring on an electrophysiologic level.

## Conclusions

With this case study, we aim to bring awareness to the sometimes-elusive presentation of PVS. In doing so, we hope to prevent the disease from being overlooked as noncardiogenic syncope or even epilepsy. Additionally, we hope to foster a more detailed evaluation of disease pathophysiology so that the medical community can better make sense of how its underlying mechanism relates to its clinical presentation. 
